# Milder outcomes of SARS-CoV-2 genetically confirmed reinfections compared to primary infections with the delta variant: A retrospective case-control study

**DOI:** 10.3389/fmed.2022.962653

**Published:** 2022-10-06

**Authors:** Alen Suljič, Maja Sočan, Maja Mrzel, Maja M. Lunar, Miša Korva, Alenka Štorman, Katarina Prosenc, Sandra Janežič, Tjaša Žohar-Čretnik, Tina Zupanič, Mario Poljak, Tatjana Avšič-Županc

**Affiliations:** ^1^Faculty of Medicine, Institute of Microbiology and Immunology, University of Ljubljana, Ljubljana, Slovenia; ^2^National Institute of Public Health, Ljubljana, Slovenia; ^3^National Laboratory of Health, Environment, and Food, Maribor, Slovenia

**Keywords:** SARS-CoV-2, COVID-19, reinfection, Delta variant, NGS, genetically confirmed variant, protective immunity, severity

## Abstract

**Background:**

SARS-CoV-2 infection does not confer long immunity. However, studies suggest that prior infection is associated with lower risk of reinfection and milder outcomes of recurrent infections. The aims of this retrospective observational case-control study were to describe the clinical and molecular characteristics of genetically confirmed Delta reinfection cases and to assess the potential protective role of preceding infection on the severity of reinfection.

**Methods:**

We used next generation sequencing (NGS) to explore if cases with two positive real time RT-PCR tests > 90 days apart were infected with a different SARS-CoV-2 variant. Cases with confirmed reinfection between August 1st and October 31st, 2021 (the Delta wave) in Slovenia were matched 1:4 by age, sex and timeframe (week of positive test) with individuals with primary infection. Sociodemographic and epidemiologic data, vaccination status, and data on hospitalization and outcome of infection were retrieved from several centralized and standardized national databases. Additional epidemiologic surveys were performed on a limited number of cases and controls.

**Results:**

We identified 628 cases of genetically confirmed reinfection during the study period and matched them with 2,512 control subjects with Delta primary infection. Primary infections in individuals with reinfection were mainly caused by B.1.258.17 (51.1%), followed by B.1.1.7 (15.1%) and reinfection was detected on average 271 days after primary infection (range 101–477 days). Our results show a substantially lower probability of hospitalization in cases with reinfection compared with controls (OR: 0.21, *p* = 0.017), but no significant difference was observed in intensive care unit admission and deaths. We observed a significantly lower proportion of vaccinated individuals among cases compared to controls (4.5% vs. 28.2%), suggesting that hybrid immunity leads to lower probability of reinfection. Detailed analysis of the temporal distribution of variants, responsible for reinfections, showed no significant differences in reinfection potential.

**Conclusion:**

Reinfection with the SARS-CoV-2 Delta variant resulted in fewer hospitalizations compared to the primary Delta infection, suggesting that primary infection may, to some extent, produce at least short lasting protective immunity. This study provides additional insight into the reinfection dynamics that may allow appropriate public health measures to be taken in subsequent waves of the COVID-19 pandemic.

## Introduction

The ongoing coronavirus disease 19 (COVID-19) pandemic has an extensive societal and economic impact (1). With each new wave of the pandemic, healthcare systems have faced large numbers of patients requiring hospitalization and intensive care unit (ICU) treatment (2) and there is growing evidence of the long-term consequences of SARS-CoV-2 infection (3). Moreover, overcoming SARS-CoV-2 infection does not provide long immunity (4). Epidemiological studies (population-based cohort studies and case-control studies) indicated that prior SARS-CoV-2 infection was associated with a significantly lower risk of reinfection over a period of 7 months to more than 1 year and for variants circulating in communities at the time of the study (5–9).

The majority of cases with reinfection had similar disease severity comparable to the first infection or had milder disease in the second episode. Cases of reinfection with SARS-CoV-2 with an adverse outcome have been described (10). The question arises regarding to what extent previously naturally acquired immunity protects against reinfection with different variants of SARS-CoV-2 and how waning immunity contributes to the frequency of reinfection (8). Different levels of exposure derived from socioeconomic determinants, occupation, living in institutional settings, differences in demographics, and comorbidities contribute to the risk of reinfection (11). The continued emergence of SARS-CoV-2 variants with higher transmissibility, immune escape, and altered pathogenicity are drivers of an increasing number of reinfections as of November 2021 (12).

Natural immunity following primary SARS-CoV-2 infection provided more sustained protection against the B.1.617.2 (Delta) variant than vaccine-mediated immunity (13). Recent studies have shown that vaccine-mediated immunity wanes after 6 months, with efficacy against the Delta variant declining rapidly after only 90 days (13, 14). Planas et al. found reduced neutralization of the SARS-CoV-2 Delta variant in comparison to previous strains (15). There was an indication toward increased severity associated with B.1.617.2 and prolonged viable viral shedding with more severe symptoms than in those infected with non-Delta variants (16, 17). Recent studies have also shown that the Delta variant was associated with an increased risk of hospitalization, ICU admission, oxygen requirement, and death (18).

In May 2021, the Delta variant was detected sporadically in Slovenia, with increasing frequency. Starting in mid-July 2021, the SARS-CoV-2 Delta variant became practically the only variant identified during national routine weekly SARS-CoV-2 genomic surveillance in Slovenia (19, 20). The increasing number of reinfections during the Delta wave provided unique opportunity to investigate the impact of pre–Delta variant SARS-CoV-2 infection on the severity of reinfection compared to primary Delta variant infections. Thus, the aims of this study were to describe the clinical and molecular characteristics of genetically confirmed Delta reinfection cases and to assess the potential protective role of preceding SARS-CoV-2 infection on the severity of reinfection.

## Materials and methods

### Design and eligibility criteria

We conducted a retrospective observational case-control study in residents of Slovenia with PCR-confirmed SARS-CoV-2 reinfection (cases) and primary infection (controls) between August 1st and October 31st, 2021 (the Delta wave).

### Sources of data

Four national health administrative data sources collecting individual health information data were used.

#### National COVID-19 database

Data were extracted from the National COVID-19 Database, which is part of the National Notifiable Communicable Diseases Database. The database covers all SARS-CoV-2 cases (symptomatic and asymptomatic) in Slovenia. The National COVID-19 Database is linked to the Central Registry of Patient Data, the Central Registry of Spatial Units, and the Register of Health Workers to obtain socio-demographic and health-related data. Data extracted from National COVID-19 Database were age (in years), sex, being a healthcare worker, living in a long-term care facility, date of first confirmed infection and date of reinfection, and time interval between initial infection and reinfection (in days). For a limited number of cases, epidemiological surveys were completed with additional data available; that is, being symptomatic or asymptomatic at the time of a confirmatory real-time RT-PCR test, having an epidemiological link to a confirmed case, and which clinical symptoms were present or absent (fever, cough, sore throat, breathing difficulties, anosmia/ageusia, headache, myalgia, and arthralgia).

##### Inclusion criteria: Cases

A case was defined according to the following criteria: (i) two laboratory-confirmed SARS-CoV-2 episodes at least 90 days apart as registered in Slovenia, (ii) the second episode (i.e., reinfection) between August 1st and October 31st, 2021, (iii) samples of both episodes (i.e., primary infection and reinfection) were SARS-CoV-2–positive by a real-time RT-PCR assay and were available for sequencing, and (iv) genomic sequencing of paired samples was performed, yielding two distinct variants of SARS-CoV-2. These strict criteria were chosen to provide high-quality laboratory evidence of reinfection and to exclude potential long-term shedding. The study period of August–October 2021 was chosen to eliminate variant bias because Delta was the only variant circulating in Slovenia at that time. After limiting the cases to these criteria, 628 cases were identified.

##### Inclusion criteria: Controls

The control group consisted of individuals matched for age, sex, and timeframe (week of positive test) with real-time RT-PCR–confirmed SARS-CoV-2 Delta virus primary infection in the same period of time as reinfection occurred in cases (August 1st to October 31st, 2021). If an exact age match was not possible, a ± 2-year tolerance was allowed. When multiple controls were available, random matching was performed. Repetition of controls was not allowed. For every case, four controls were identified (i.e., 2,512 controls altogether).

#### National vaccination register

We obtained data on vaccination against COVID-19 from the eRCO national vaccination register (Slovenian: *Elektronski register cepljenih oseb* “Electronic Register of Vaccinated Persons”). The data extracted from the eRCO register were the date of the vaccination and the vaccine used.

Cases and controls were classified as fully vaccinated if they had received one dose of Jcovden vaccine or both doses of the two-dose schedule vaccines (mRNA vaccine: Comirnaty or Spikevax, vector vaccine: Vaxzevria) at least 14 days before reinfection (cases) or primary infection (controls). Partially vaccinated cases and controls received one dose of two-dose COVID-19 vaccines at least 14 days before confirmation of reinfection (cases) or primary SARS-CoV-2 infection (controls). Beyond that, as partially vaccinated we also counted persons that had received both doses of two-dose vector or mRNA vaccines but for whom less than 14 days had elapsed between vaccination and a positive PCR test for SARS-CoV-2.

#### National registry of hospitalizations

Hospitalization data were obtained from the eSBO national registry of hospitalizations (Slovenian: *Elektronski sistem bolnišničnih obravnav* “Electronic Registry of Hospitalizations”). Temporally associated admissions (14 days before and 14 days after positive PCR) to acute care hospitals were analyzed. Data collected from eSBO were main discharge and additional diagnoses, duration of hospitalization (in days), intensive care treatment (in hours), and outcome (discharge, death). By definition, COVID-19 was the cause of hospitalization if classified as the main discharge diagnosis (ICD-10 classification U07.1) or if the main discharge diagnosis was viral pneumonia caused by SARS-CoV-2.

#### National registry of deceased persons

The National Registry of COVID-19 Cases is regularly updated with data from the National Registry of Deceased Persons. Death is attributed to COVID-19 according to the WHO definition (i.e., death within 28 days after laboratory-confirmed SARS-CoV-2 infection).

Individual data in the national registries were linked by a unique personal identification number. The National COVID-19 Database, eRCO, eSBO, and the National Registry of Deceased Persons are managed by the National Institute of Public Health (NIPH) of Slovenia.

#### GISAID repository

To determine the prevalence of SARS-CoV-2 lineages observed in the Slovenian population, we accessed the GISAID global database^1^ and extracted the corresponding prevalence for each lineage detected. We calculated the percentage of the population based on the data for the entire country. This information was used to compare the prevalence of lineages detected in the primary infection of cases to the prevalence in Slovenian population. Any major deviations in prevalence could indicate a bias toward a particular lineage with regard to reinfection potential. This comparison is only possible if the assumption of representativeness of the national surveillance ability to reliably detect circulating lineages is not violated. In other words, we want to be certain (or at least know the limits of certainty) that national data on lineage presence in sequenced samples could be generalized to the entire population of Slovenia. According to the ECDC guidelines (21), our national SARS-CoV-2 surveillance strategy allowed us to detect and characterize lineages with a prevalence of less than 1%.

### Laboratory analysis

#### Sample collection

Nasopharyngeal swab samples were collected as part of routine testing for SARS-CoV-2 at the Institute of Microbiology and Immunology, Faculty of Medicine, University of Ljubljana and the National Laboratory of Health, Environment, and Food of the Republic of Slovenia. After the identification of possible cases of reinfection, all samples that had not already been sequenced as a part of routine weekly surveillance for SARS-CoV-2 variants were collected by laboratory personnel for retrospective sequencing.

#### Library preparation and next generation sequencing sequencing

RNA was extracted from 300 μl of nasopharyngeal swab samples using Maelstrom 9600 (TanBead Inc., Taoyuan, Taiwan), according to the manufacturer’s instructions. PCR amplicons were prepared in accordance with the ARTIC V2 and V3 RT-PCR protocol [nCoV-2019 sequencing protocol v2 (GunIt)].^2^ PCR amplicon size was inspected on 2% agarose gel. DNA concentration was measured with the Qubit dsDNA High Sensitivity assay kit on Qubit 3.0 (both Thermo Fisher Scientific, Waltham, MA, USA). We prepared NGS libraries of amplicons using the Nextera XT library preparation kit (Illumina, San Diego, CA, USA), according to the vendor’s instructions. The concentrations of NGS libraries were measured using the Qubit dsDNA High Sensitivity assay on a Qubit 3.0 instrument (Thermo Fisher Scientific). The fragment sizes were analyzed using the Agilent HS DNA Kit on the Bioanalyzer 2100 (both Agilent Technologies, Santa Clara, CA, USA). Prepared samples were sequenced using the MiSeq Reagent Kit v3 (600 cycles) on the MiSeq Sequencer, the NextSeq 500/550 High Output Kit v2.5 (300 cycles) on the NextSeq 550, or NovaSeq 6000.

### Bioinformatic analysis

Initially, we trimmed the raw reads obtained from the Illumina sequencers using BBDuk, which is part of the BBTools program package (22). The quality of the raw reads and the quality of the trimming procedure were evaluated with FastQC (23). We mapped the trimmed reads to the Wuhan-Hu-1 isolate reference genome (NCBI accession number NC_045512.2) using BWA-MEM with default settings (24). Mapped reads were subsequently transformed into an appropriate form using Samtools (25). This process included exporting the mapping data to bam files, sorting, mate-flagging, duplicate-marking, and indexing of the mapping data. Samtools was also used for coverage depth calculations. A consensus sequence was generated using iVAR (26). We set the minimum quality threshold to a factor of 10, the minimum depth for calling consensus to 10 reads, and the minimum frequency threshold to 0.5 (consensus was called when 50% of the reads agreed on a particular base). Lineage assignment was performed using the Phylogenetic Assignment of Named Global Outbreak Lineages (Pangolin), which implements the dynamic nomenclature of SARS-Cov-2 lineages (27). All sequences have been deposited in the GISAID repository and are available for further analyses (Supplementary material).

### Statistical methods

Data analysis was performed using R statistical software (version 4.1.3, R Foundation for Statistical Computing, Vienna, Austria). To assess the normality of the data distributions, we used Q–Q plots and the Shapiro–Wilk test. Differences in the number of nursing home residents between groups (cases vs. controls) were evaluated using Fisher’s exact test, and differences in the number of healthcare workers between groups were evaluated using Pearson’s chi-squared test with Yates’ continuity correction. The differences in reported symptoms and vaccination status were evaluated with a two-proportions *z*-test. For the effect size assessment between two proportions, we opted for Cohen’s *h* effect size. The odds ratio between groups was calculated using Fisher’s exact test for count data and, when necessary, Haldane’s correction on zero values was applied. Differences in the number of asymptomatic disease courses between vaccinated and unvaccinated individuals were evaluated with Fisher’s exact test. The difference in time intervals between first infection and reinfection was assessed with ANOVA. The threshold for statistical significance was set at *p* < 0.05 in all cases.

The study followed the Strengthening the Reporting of Observational Studies in Epidemiology (STROBE) reporting guideline for case-control studies.

## Results

From the beginning of the COVID-19 epidemic in Slovenia (the first case was identified on March 4th, 2020) to October 31st, 2021, there were 333,959 Slovenian residents with a positive RT-PCR test (320,428 persons) or rapid antigen test (RAT) (13,531 persons). According to the national case definition, RAT was accepted as a confirmatory test for a short period of time (from December 21st, 2020 to February 12th, 2021).

The 320,428 RT-PCR–positive individuals included 318,805 individuals with single confirmed SARS-CoV-2 infection and 1,623 individuals with possible reinfection; that is, with two real-time RT-PCR–positive tests at least 90 days apart. Whole genome sequencing (WGS) confirmed two distinct variants of the SARS-CoV-2 virus in the first and second samples in 660 cases, including 32 reinfections occurring before the Delta wave. Finally, 628 persons with the first infection before the Delta wave and reinfection during the Delta wave were included in the study. In 963 cases with possible reinfection, one or both samples were unavailable for WGS, WGS was unsuccessful, or individuals were found to have a prolonged infection (the same SARS-CoV-2 variant was detected in both samples).

### Demographics, source of infection, and clinical presentation

**FIGURE 1:**
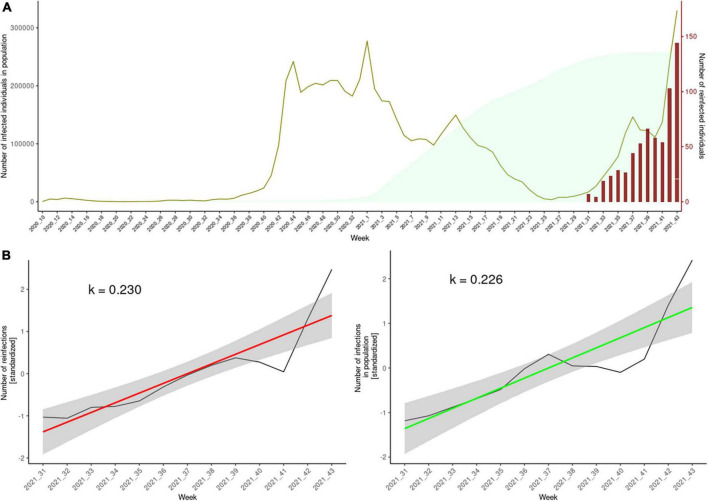
Panel **(A)** of this composite plot shows the weekly cumulative total of people eligible for reinfection (light green), the weekly number of positive SARS-CoV-2 tests times 20 (olive), and the number of confirmed reinfections (red). The y-axis on the right side of the plot corresponds to the number of reinfected individuals. The y-axis on the left side corresponds to the number of individuals eligible for reinfection and the number of weekly infections. In order to simultaneously present the two in the same plot, we multiplied the number of weekly infections by 20 (to illustrate, the peak in olive green in the first week of 2021 represent ∼ 15,000 individuals). The cumulative plot, which represents the pool of potential reinfections, is presented with a 90-day lag, according to the ECDC definition criterion for reinfection. Panel **(B)** depicts the standardized rise of weekly reinfection numbers (red regression line) and the weekly number of positive tests (green regression line).

As shown in Table 1, we identified 382 (60.8%) females and 246 (39.2%) males with genetically confirmed reinfection (i.e., cases) with Delta in the study period, from 4 to 92 years of age, with the majority of cases (75.2%) in the 20–49 age group. We compared the proportion of cases with the proportion of the population to determine possible differences in reinfection potential. Initial infections in individuals with reinfection were mainly caused by B.1.258.17 (51.1%), followed by B.1.1.7 (15.1%; Table 2). The study period was selected at the beginning of the Delta wave in Slovenia, and therefore the distribution of cases (and matched controls) according to the week in which reinfection occurred was skewed to the right, as seen in Figures 1A,B. The cases and matched controls did not differ in the proportion of healthcare workers or nursing home residents (Table 1). The proportion for being asymptomatic was higher among cases (*p* = 0.004), and those cases that were symptomatic had on average statistically significant fewer symptoms compared to controls (*p* = 0.02). A comparison of clinical data showed that cases had statistically significantly lower proportions of loss of smell and taste and proportions of accompanying fever (*p* = 0.005 and *p* < 0.001, respectively) and statistically significantly higher proportions of headache and sore throat (*p* = 0.03 and *p* = 0.03, respectively) compared to controls (Table 1). Cases and controls had the same proportion of known sources of infection; however, we observed a higher proportion of infections from a family member among the controls (*p* = 0.01).

**TABLE 1:** Matching and non-matching variables in cases (individuals with Delta SARS-CoV-2 reinfection) and controls (individuals with initial Delta SARS-CoV-2 infection).

	Cases, *n* = 628		Controls, *n* = 2,512		*P*-value	Cohen’s *h* (effect size)
**Demographics**						
**Sex**						
Female, *n* (%)	382	(60.8)	1,528	(60.8)	–	–
Male, *n* (%)	246	(39.2)	984	(39.2)	–	–
Age (years), mean (range)	34	(4–91)	33	(4–93)	–	–
0–9, *n* (%)	10	(1.6)	40	(1.6)	–	–
10–19, *n* (%)	76	(12.1)	304	(12.1)		
20–29, *n* (%)	149	(23.7)	596	(23.7)		
30–39, *n* (%)	182	(29.0)	728	(29.0)		
40–49, *n* (%)	141	(22.5)	564	(22.5)		
50–59, *n* (%)	51	(8.1)	204	(8.1)		
60–69, *n* (%)	10	(1.6)	40	(1.6)		
70–79, *n* (%)	3	(0.5)	12	(0.5)		
80 + , *n* (%)	6	(1.0)	24	(1.0)		
Healthcare worker, *n* (%)	27	(4.3)	87	(3.5)	0.4	–
Nursing home resident, *n* (%)	5	(0.8)	10	(0.4)	0.2	–
Teachers (pre-, primary, secondary schools), *n* (%)	59	(9.4)	102	(4.1)	<0.001	0.24
**EPI survey data**						
Asymptomatic course, *n* (%)	27/371	(7.3)	63/1,610	(3.9)	0.004	0.15
Fever, *n* (%)	113/249	(45.4)	796/1,192	(66.8)	<0.001	0.43
Loss of taste and smell, *n* (%)	61/249	(24.5)	395/1,194	(33.1)	0.005	0.19
Sore throat, *n* (%)	75/249	(30.1)	285/1,194	(23.9)	0.02	0.14
Headache, *n* (%)	76/249	(30.5)	292/1,194	(24.5)	0.03	0.14
Muscle and joint pain, *n* (%)	47/249	(18.9)	195/1,194	(16.3)	0.4	–
Cough, *n* (%)	141/249	(56.6)	694/1,194	(58.1)	0.7	–
Difficulty breathing, *n* (%)	9/249	(3.6)	39/1,164	(3.4)	0.9	–
Shortness of breath, *n* (%)	2/249	(0.8)	21/1,175	(1.8)	0.4	–
ARDS, *n* (%)	0/249	(0)	3/1,191	(0.3)	0.9	–
No. of reported symptoms, mean/*n*, (*SD*)	2.1/249	(1.16)	2.3/1,194	(1.17)	0.02	0.15
Epi. link/Contact with a confirmed case, *n* (Yes)	165/273	(60.4)	771/1,195	(64.5)	0.2	–
Most probable source of infection: family, household, *n* (%)	92/203	(45.3)	516/942	(51.8)	0.01	0.19
**Vaccination**						
Unvaccinated, *n* (%)	575	(91.6)	1,753	(69.8)	<0.001	0.57
Partial, *n* (%)	25	(4.0)	47	(1.9)	0.001	0.13
Full, *n* (%)	28	(4.5)	708	(28.2)	<0.001	0.69
Boost, *n* (%)	0	(0)	4	(0.2)	0.7	–

ARDS, acute respiratory distress syndrome.

**TABLE 2:** Genomic variant composition of primary infections in individuals with the Delta variant reinfection.

Variant	*n*	Sample, %	Population, %	Ratio Sample /Population
B.1.258.17	321	51.1	38.7	1.3
B.1.1.7	95	15.1	42.9	0.4
B.1.258	64	10.2	3.6	2.8
B.1.1.70	45	7.2	3.5	2.1
B.1.160	36	5.7	4.2	1.4
B.1.149	11	1.8	1.1	1.6
B.1.1	9	1.4	1.4	1.1
B.1.146	8	1.3	0.5	2.8
C.35	8	1.3	0.7	1.8
B.1.177	6	1.0	0.5	1.9
B.1	3	0.5	1.7	0.3
B.1.236	3	0.5	0.4	1.4
B.1.1.39	2	0.3	0.0	8.1
B.1.160.14	2	0.3	0.0	32.3
B.1.221	2	0.3	0.2	1.5
C.16	2	0.3	0.1	5.4
A	1	0.2	0.0	32.3
AP.1	1	0.2	0.0	32.3
B.1.1.58	1	0.2	0.4	0.4
B.1.177.28	1	0.2	0.2	0.7
B.1.224	1	0.2	0.0	32.3
B.1.243	1	0.2	0.0	32.3
B.1.36.1	1	0.2	0.0	10.8
B.1.36.23	1	0.2	0.0	5.4
B.1.389	1	0.2	0.0	10.8
B.1.94	1	0.2	0.0	32.3
Q.1	1	0.2	0.0	32.3

The differences are expressed as ratios between percentages. A ratio lower than 1 indicates underrepresentation of the lineage in our sample (cases) in comparison to the population, and a ratio greater than 1 shows overrepresentation of the lineage in our sample in comparison to the population.

### Vaccination

The vaccination status of cases and controls is presented in Table 1. Most cases were unvaccinated (575 cases, 91.6%) compared to statistically significantly fewer unvaccinated controls (1,753, 69.8%). Only 28 (4.5%) cases were fully vaccinated with Comirnaty (14 persons), Vaxzevria (4 persons) or Jcovden (10 persons). Among fully vaccinated controls 345 received Comirnaty, 191 Vaxzevria, 138 Jcovden, 32 Spikevax and 2 received a combination of Vaxzevria/Comirnaty. We observed statistically significant differences in vaccination status between groups. A significantly higher proportion of unvaccinated patients was observed among cases, with a large effect size. Furthermore, a significant difference was observed in the ratio of fully vaccinated patients between groups, with a lower proportion of cases vaccinated with both doses (Table 1). We found no statistically significant differences in asymptomatic disease course in relation to vaccination status (*p* = 0.16).

### Disease severity

A statistical analysis of severity indicators (hospitalizations, ICU admissions, and death in the first 28 days after RT-PCR positivity) showed lower odds for hospitalizations in cases (OR 0.21, CI 0.05–0.86, *p*-value = 0.02), but not for ICU admissions and deaths (Table 3). The hospitalized cases were one male and one female, both unvaccinated. The first case had reinfection 5 months after initial infection, admitted to the ICU, and mechanically ventilated. The second case had reinfection 7 months after initial infection and hospitalized for both episodes, although no ICU or mechanical ventilation was needed. Six deaths were recorded in the control group and none in the cases with reinfection. The deceased patients were four females and two males age 51 to 92 years, who died 1 to 26 days after the RT-PCR–positive test result. Four of them died in the hospital, and only one needed ICU treatment with mechanical ventilation. The other two died after discharge, but both had severe underlying disease (cancer and diabetes, respectively).

**TABLE 3:** Hospitalizations, ICU admissions, and deaths in cases and controls.

Severity	Cases, *n* (%)		Controls, *n* (%)		OR (95% CI)	*P*-value	Cohen’s *h* (effect size)
Hospitalization	2	(0.3)	38	(1.5)	0.21(0.05–0.86)	**0.02**	0.11
ICU	1	(0.2)	5	(0.2)	0.80(0.09–6.86)	1	–
Death	0	(0.0)	6	(0.2)	0.31(0.02–5.45)	0.6	–

### Temporal distribution of the timing of both SARS-CoV-2 episodes in cases

**FIGURE 2:**
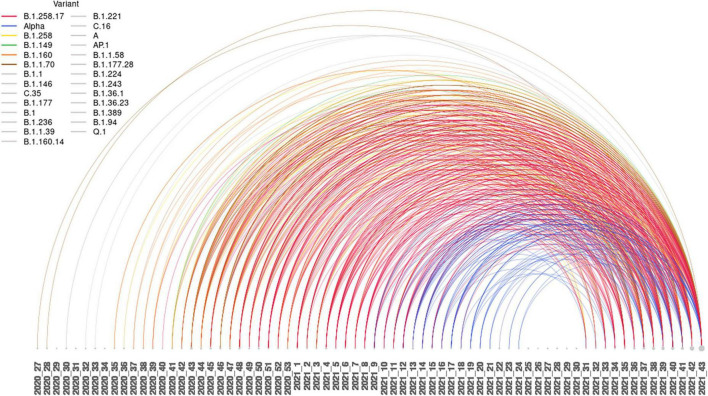
Arc plot of reinfection intervals between the first and second SARS-CoV-2 episodes. The lines are color coded for the six most represented variants.

**FIGURE 3:**
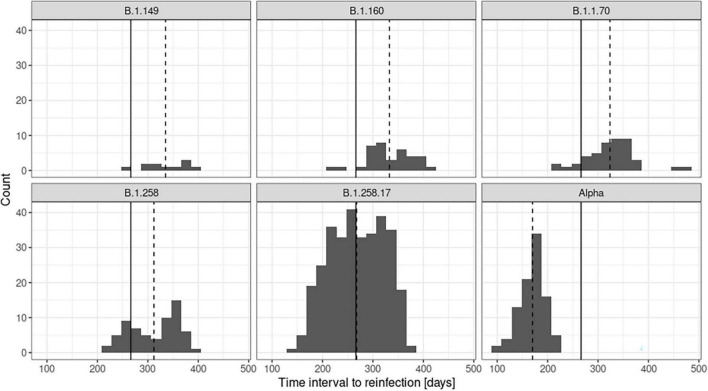
Time intervals between first infection and reinfection for the most common variants observed in primary infection in cases. The global mean of the time intervals between infections across all variants is shown as a solid line, and the mean interval for each variant is shown as a dashed line.

One can observe a heterogeneous composition of variants responsible for the first infection, or, from another perspective, the absence of any clusters that would indicate a bias toward a particular variant being more susceptible to reinfection with the Delta variant. Figure 2 shows the relational data for the cases. The time elapsed between first and second infection was a minimum of 101 days and a maximum of 477 days, on average 271 days, as presented in Figure 2. The variant distribution of the first SARS-CoV-2 episode of the cases is presented in Table 2. The frequencies of variants of cases are shown next to the population prevalence of each variant in Slovenia until August 1st, 2021 according to GISAID. The main finding is a notably lower percentage of the Alpha variant in the sample compared to the percentage in the population (15.1% vs. 42.9%). We observed an average time to reinfection of 271 days after primary infection. Figure 3 presents the distributions of time intervals between primary infection and reinfection for each of the variants that occurred in at least 10 cases. We can observe that the emergence and prevalence of the variant directly correspond to the time interval. In other words, the “older” the variant, the longer the mean interval. The most prominent result is the notably shorter intervals in the Alpha variant.

## Discussion

In this case-control study, we aimed to characterize the differences between individuals with SARS-CoV-2 reinfection and individuals first diagnosed with SARS-CoV-2, both infected with the Delta variant in the same calendar week. The main strength of this study is the rigorous genomic characterization of variants detected in paired samples from cases with reinfection using next-generation sequencing (NGS). This approach reinforces the comparison analysis and eliminates the false-positive bias that is introduced when accurate genomic assignment is not employed. The thorough analysis of patient metadata and genomic information is complemented by the use of national registry resources for all cases included and a fairly large control group matched by sex, age, and week of positive SARS-CoV-2 test, which ensures sufficient statistical power to differentiate between groups.

Analysis of demographic data showed no age difference between female and male cases, which may suggest that age does not play an important role in reinfection dynamics. However, female cases were overrepresented (61%). In Slovenia, there were more females with confirmed SARS-CoV-2 infection than males throughout the pandemic (53 and 47%, respectively; national data available from dashboard),^3^ but not as many as in the cases included in our study. The analysis showed that more cases were employed in the education and health sectors compared to the general population (Table 1). About 2% of the Slovenian general population works in each sector, whereas 9% of cases are employed in the education sector and 4% in the health sector. Because women are predominantly employed in both sectors and frequent testing for SARS-CoV-2 was mandatory in both occupational groups, this could explain the female predominance. However, we did not find a statistically significant difference in the proportion of healthcare workers and nursing home residents between cases and controls. A number of studies that exclusively enrolled healthcare workers reported lower odds ratios for reinfection and less severe disease course in this profession (5, 28, 29). We were unable to replicate this finding, most likely due to the low prevalence of reinfection (range 0.1–1.1%) and the relatively small number of healthcare workers and nursing home residents in our data (5–7, 30). Similar results have been reported for nursing home residents. Although residents were among the first to be infected during the first wave, there is no evidence of a higher risk of reinfection in this group (31).

The main finding of this study is the observed statistically significant difference in the number of hospitalizations between cases and controls (two vs. 38, respectively). The calculated odds ratio of 0.2 (CI: 0.1–0.8) suggests the protective role of prior infection (approximately five times lower odds of hospitalization at reinfection in comparison to first infection). However, due to the low numbers of hospitalization events, we were confronted with a relatively broad confidence interval, which does not allow us to draw a firm conclusion about the assumed protective nature of prior infection. In addition, because of the retrospective study design, we cannot exclude survival bias, which may have contributed to this observation. Six deaths were observed among control subjects in the study and none among cases, which could also support this hypothesis, but the numbers are too small to draw any conclusions. Data on COVID-19 hospitalization encompass the entire spectrum of causes and populations in exposure to SARS-CoV-2, however in our study, we limited the hospitalization rates specifically to “COVID-19” or “viral pneumonia” diagnoses 14 days before and 14 days after positive SARS-CoV-2 PCR. This was probably the reason for a relatively small number of hospitalization events recorded, however, there is also a possibility that we introduced a small measure of sample selection bias. Although we took some measures to prevent such eventuality (sufficiently large control group, thorough demographic characterization of chosen sample), we cannot reliably and completely eliminate sample selection bias. For a limited number of cases and controls, additional information was made available from surveys conducted by trained epidemiologists. The most prominent finding was the observed higher proportion of SARS-CoV-2 RT-PCR–positive individuals that reported having had fever on initial infection with the Delta variant compared to persons with reinfection with this variant (67% vs. 45%, respectively). Furthermore, we identified a higher proportion of reported asymptomatic cases in comparison to the control group (7% vs. 4%, respectively), and a significant lower proportion of cases reporting loss of smell and taste in comparison to the control group (24% vs. 33%, respectively). This result could indicate a protective nature of prior infection in the reinfection course. However, we also observed a significantly higher proportion of reported sore throat in the case group (30% vs. 24% in the control group) and a significantly higher proportion of headache in the case group (31% vs. 25% in the control group). The complex interplay of immune system response, virus characteristics, and the social conduct of the individuals makes the characterization of symptomatic profiles difficult (32). To address this issue would require a more focused study to further elucidate the factors that contribute to a specific symptom profile in each group.

Another noteworthy observation was a statistically significant difference in vaccination status between groups. The case group had a significantly higher proportion of unvaccinated individuals in comparison to the control group (91.6% vs. 69.8%, respectively). This could be explained by individuals being less inclined to vaccinate after having already experienced SARS-CoV-2 infection (33). Natural infection in combination with vaccination (i.e., hybrid immunity) offers better protection against reinfection. This finding was supported by a large observational study in Israel, which reported that SARS-CoV-2–naive individuals that received two doses of the Comirnaty vaccine were six to 13 times more likely to become infected with Delta than patients that had previously experienced infection (14). The reported vaccination imbalance lacks the background information that would set the difference in a broader perspective. We cannot pin this discrepancy to the most common demographic factors, such as age or gender, due to age and gender matched study design. On the other hand, we cannot with certainty exclude the presence or frequency of comorbidities as a confounding factor. The vaccination status is determined by the plethora of complex factors. The COVID-19 vaccine acceptance depends on perceived risk of disease, level of trust in the vaccine, in the delivery system and the recommendations given by health authorities and barriers related to geographic accessibility and, availability of vaccination services (34).

Analysis of the temporal distribution of variants causing initial infection did not reveal any obvious patterns that would suggest a bias for reinfections toward an identified variant in first infection. As expected, we observed that the variant mean time interval to reinfection directly corresponded to the emergence of the variant: the “older” the variant, the longer its mean time interval to reinfection. This is most likely the reason why we observed notably shorter intervals (≈ 170 days) for the Alpha variant, a major variant that preceded the Delta wave studied here. However, the immune escape can also contribute to this effect. The lineage B.1.258.17 already harbored some specific spike mutations, such as del69_70 and N439K that have been reported to be associated with increased infectivity (35), reduction of binding affinity for the ACE2 receptor and reduced neutralizing activity of some monoclonal and polyclonal antibodies (36). Alpha exhibited additional deletion in RBD domain – del144/144, which was reported causing a fourfold reduction in neutralization titer (37). In a comparison between wild-type virus isolate harboring D614G mutation, Alpha and Delta, (38), reported that Alpha virus isolate was 2.3-fold less sensitive to the neutralizing antibodies than WT_D614G, and that Delta was 5.7-fold less sensitive to the neutralizing antibodies. On the other hand, while examining the variant composition of the SARS-CoV-2 initial episode of cases and comparing it to the prevalence of variants as indicated by GISAID until the study period, we detected a significant deviation in percentages of the Alpha variant (19, 20). It appears that the Alpha variant was underrepresented in our reinfection cases, which could indicate a higher protective capacity of the Alpha variant against Delta or could be due to a shorter time interval and immunity that was still present at the time of our study period but began to wane thereafter. Although there is a notable difference between the presence of the Alpha variant in our cases and in the population, we speculate that, if we extended the time interval to include the rest of the year 2021, we would see a rise in the percentage of Alpha as first infection in reinfection cases.

Even though this study was designed to minimize SARS-CoV-2 variant bias and set strict criteria for classification of reinfection, it has some limitations. First, cases were not randomly selected, but were chosen based on the availability of samples and finally generation of the SARS-CoV-2 genetic sequence. This eventuality exposes the results of our study to the potential of sample selection bias. However, because we included the samples from all laboratories in the country that performed SARS-CoV-2 testing, we assumed a representative sample of the population. Even though some samples were unavailable due to technical reasons, we believe that this disruption did not introduce a systemic bias.

Next, data were obtained from national repositories, but unfortunately, not all potentially interesting data were available. For example, to study the severity of the disease, it would be of great benefit if the available data were supplemented with an exhaustive medical history of the individuals investigated. This information would enable us to further refine our findings and perhaps reveal a subset of individuals with specific comorbidities that are more susceptible for reinfection. There is a higher proportion of asymptomatic cases compared to controls. However, reporting these cases was difficult because many asymptomatic infections may have been missed and underestimated, possibly because of the reluctance to screen the individuals selected here as controls. Finally, no additional testing was performed, thus we cannot completely exclude the possibility of some unrecognized reinfections in the selected controls. This could be avoided to some extent by additional serological testing, which was not performed because of the retrospective nature of this study.

Naturally acquired immunity to SARS-CoV-2 is not long-lasting and vanished within a few months, as does immunity after vaccination. The results of this study confirm that a preceding infection provides some protection against reinfection with the Delta variant and reduces the severity of the disease. The number of reinfections with SARS-CoV-2 increased during Omicron wave and is likely to increase in the future. For adapting timely and appropriate public health response, it is important to closely track the evolution of variants and the impact on previously infected individuals.

## Data availability statement

The datasets presented in this study can be found in online repositories. The names of the repository/repositories and accession number(s) can be found below: https://www.gisaid.org/, EPI_SET_220901qg (https://epicov.org/epi3/epi_set/220901qg?main=true).

## Ethics statement

The studies involving human participants were reviewed and approved by Ethics Board at National Institute of Public Health, Slovenia (consent no. 1810-92/2021-18). Data used for the study were routinely collected for health statistics or epidemiological purposes. According to the Healthcare Databases Act, patient consent is not required to collect such data. Written informed consent for participation was not required for this study in accordance with the national legislation and the institutional requirements.

## Author contributions

MS, ML, MK, MP, and TA-Ž conceived and designed the project. MK, KP, AŠ, SJ, and TŽ-Č contributed the data. AS, MM, ML, and TZ analyzed the data. AS, MS, MM, and ML drafted the manuscript. MS, MK, MP, TA-Ž, AŠ, and KP revised the manuscript. All the authors contributed to the manuscript and approved the submitted version.

## Footnotes

1
https://www.gisaid.org

2
https://www.protocols.io/view/ncov-2019-sequencing-protocol-v2-bp2l6n26rgqe/v2?version_warning=no

3
https://www.nijz.si/sl/dnevno-spremljanje-okuzb-s-sars-cov-2-COVID-19
