# Supplementary material for: Milder outcomes of SARS-CoV-2 genetically confirmed reinfections compared to primary infections with the delta variant: A retrospective case-control study
Source: Front Med (Lausanne). 2022 Oct 6;9:962653. doi: 10.3389/fmed.2022.962653 (PMC9582599; doi:10.3389/fmed.2022.962653)
Supplement: Supplementary file 1 [file Data_Sheet_1.pdf]

SUPPLEMENTAL TABLE

Data Availability

GISAID Identifier: EPI_SET_220901qg
doi: 10.55876/gis8.220901qg

All genome sequences and associated metadata in this dataset are published in GISAID's EpiCoV database. To view the contributors of each individual sequence with details such as accession number, Virus name, Collection date, Originating Lab and Submitting Lab and the list of Authors, visit 10.55876/gis8.220901qg

Data Snapshot

- EPI_SET_220901qg is composed of 624 individual genome sequences.
- The collection dates range from 2020-07-03 to 2021-08-16;
- Data were collected in 1 countries and territories;
- All sequences in this dataset are compared relative to hCoV-19/Wuhan/WIV04/2019 (WIV04), the official reference sequence employed by GISAID (EPI_ISL_402124). Learn more at https://gisaid.org/WIV04.
